# Protein Misfolding and Neurodegenerative Diseases

**DOI:** 10.1155/2014/217371

**Published:** 2014-03-31

**Authors:** Alessio Cardinale, Roberto Chiesa, Michael Sierks

**Affiliations:** ^1^Laboratory of Molecular and Cellular Neurobiology, IRCCS San Raffaele Pisana, Via di Val Cannuta, 247 00166 Rome, Italy; ^2^Laboratory of Prion Neurobiology, Department of Neuroscience, Istituto di Ricerche Farmacologiche Mario Negri, Via G. La Masa, 19 20156 Milan, Italy; ^3^Department of Chemical Engineering, Arizona State University, P.O. Box 876106, Tempe, AZ 85287-6106, USA

This special issue includes fifteen reviews and two original research articles by leading scientists in the fields of neuropathology, biochemistry, and cell biology, dealing with the role of protein aggregation and prion-like propagation of protein misfolding in neurodegenerative diseases.

In the review article “*Breaking the code of amyloid-*β* oligomers,*” available at the following link: http://www.hindawi.com/journals/ijcb/2013/950783/, S. E. Lesnè outlines the “oligomeric” view of the amyloid hypothesis in Alzheimer's disease (AD), discussing how structurally different amyloid-*β* (A*β*) oligomers may contribute to the pathogenesis, and the controversial role of the prion protein (PrP) in A*β* toxicity. He stresses the need to thoroughly characterize the oligomeric A*β* assemblies for dissecting the disease mechanisms and designing specific, effective therapies.

Tau oligomers may also play an important neurotoxic role in AD. In the research article “*Trimeric tau is toxic to human neuronal cells at low nanomolar concentrations,*” available at the following link: http://www.hindawi.com/journals/ijcb/2013/260787/, H. Tian et al. show that two nonphosphorylated human recombinant tau splice variants are neurotoxic at low nanomolar concentrations. They provide evidence that trimeric but not monomeric or dimeric tau is responsible for the toxicity. In the review article “*The innate immune system in Alzheimer's disease,*” available at the following link: http://www.hindawi.com/journals/ijcb/2013/576383/, A. Boutajangout and T. Wisniewski focus on the potential roles of the triggering receptor expressed on myeloid cells 2 protein (TREM2) and Toll-like receptors (TLRs) in AD. They give an overview of TREM2 functions and its involvement in phagocytic and anti-inflammatory pathways. They also review the critical roles of TLR4 and 9 in the innate immune response, the interplay of these pattern recognition receptors, and highlight the importance of microglia-mediated innate immunity in AD pathogenesis.

Several articles deal with the cellular processes involved in protein folding and quality control and how their corruption may trigger neurotoxicity. In the review article “*Disulfide bonding in neurodegenerative misfolding diseases,*” available at the following link: http://www.hindawi.com/journals/ijcb/2013/318319/, M. F. Mossuto discusses the role of disulfide bond formation; in the review article “*Role of protein misfolding and proteostasis deficiency in protein misfolding diseases and aging,*” available at the following link: http://www.hindawi.com/journals/ijcb/2013/638083/, K. Cuanalo-Contreras et al. review the involvement of the unfolded protein response (UPR), the ubiquitin proteasome system (UPS), autophagy, and aggresome formation in neurodegenerative diseases and aging. In the review article “*ER dysfunction and protein folding stress in ALS,*” available at the following link: http://www.hindawi.com/journals/ijcb/2013/674751/, S. Matus et al. specifically focus on the role of UPR in amyotrophic lateral sclerosis (ALS), and in the research article “*Early delivery of misfolded PrP from ER to lysosomes by autophagy,*” available at the following link: http://www.hindawi.com/journals/ijcb/2013/560421/, C. J. Cortes et al. provide experimental evidence of a role of autophagy in the early quality control of misfolded PrP. In the review article “*S-Nitrosation and ubiquitin-proteasome system interplay in neuromuscular disorders,*” available at the following link: http://www.hindawi.com/journals/ijcb/2014/428764/, S. Rizza et al. give an overview on the mechanisms regulating S-nitrosation and its implication in redox signaling and neurodegeneration. They provide evidence that S-nitrosation is involved in UPS and suggest links with the pathogenesis of neuromuscular disorders and neuropathies.

In the review article “*Convergence of synapses, endosomes, and prions in the biology of neurodegenerative diseases,*” available at the following link: http://www.hindawi.com/journals/ijcb/2013/141083/, G. K. Gouras critically discusses the roles played by synapses and cellular proteolytic systems in generation, accumulation, and prion-like propagation of neurodegenerative disease-specific proteins. He stresses the need to define the physiological functions of the misfolding proteins as well as to understand the cell biology of the synapses and endocytic/exocytic pathways in neurons better.

The review article “*Prion protein misfolding, strains, and neurotoxicity: an update from studies on mammalian prions*” by I. Poggiolini et al. available at the following link: http://www.hindawi.com/journals/ijcb/2013/910314/ is a comprehensive review of the prion diseases, looking at the latest information on current knowledge of the mechanisms of PrP conversion and the molecular basis of prion strains. The review article “*From prion diseases to prion-like propagation mechanisms of neurodegenerative diseases*” by I. Acquatella-Tran Van Ba et al. available at the following link: http://www.hindawi.com/journals/ijcb/2013/975832/, summarizes the history of prion diseases, from the development of the prion concept to the production of synthetic prions, and discusses recent hypotheses on the mechanisms of* de novo* prion generation. The review article “*Synaptic dysfunction in prion diseases: a trafficking problem?*” by A. Senatore et al. http://www.hindawi.com/journals/ijcb/2013/543803/, reviews recent data pointing to intracellular PrP misfolding in synaptic dysfunction and suggests a new model of synaptotoxicity that could explain the phenotypic heterogeneity of prion diseases.

The review article “*Infectivity versus seeding in neurodegenerative diseases sharing a prion-like mechanism*” by N. Fernández-Borges et al. available at the following link: http://www.hindawi.com/journals/ijcb/2013/583498/, provides a critical appraisal of the current evidence supporting prion-like mechanisms in AD, frontotemporal dementia and other tauopathies, Parkinson's disease, and ALS, discussing crucial differences between these disorders and “real” prion infections. Along this line, the review article “*Prions ex vivo: what cell culture models tell us about infectious proteins*” by S. Krauss and I. Vorberg available at the following link: http://www.hindawi.com/journals/ijcb/2013/704546/, reviews the data on the cellular propagation of different protein aggregates, demonstrating that not all the typical characteristics of prions are shared by other misfolding proteins.

In the review article “*Identification of misfolded proteins in body fluids for the diagnosis of prion diseases*” available at the following link: http://www.hindawi.com/journals/ijcb/2013/839329/, F. Properzi and M. Pocchiari give an up-to-date and comprehensive overview of the assays for detecting pathological forms of PrP in body fluids, highlighting the technological progress made in recent years. They stress the need to validate these diagnostic tools in blood samples and the importance of understanding prion metabolism in blood for effective diagnosis. The review article “*Small-molecule theranostic probes: a promising future in neurodegenerative diseases*” by S. Aulíc et al. available at the following link: http://www.hindawi.com/journals/ijcb/2013/150952/, reviews the potential diagnostic and therapeutic activity of small molecules that bind and influence the aggregation of several misfolding proteins, and the review article “*Gene-based antibody strategies for prion diseases*” by A. Cardinale and S. Biocca available at the following link: http://www.hindawi.com/journals/ijcb/2013/710406/, presents an overview of the application of intracellular antibody technology (intrabodies) in prion diseases. They concisely review the concept of intrabodies and provide recent information on* in vitro* and* in vivo* studies. They stress the importance of targeting the actual neurotoxic species in prion diseases and improving the* in vivo* stability and efficacy of vectored antiprion antibody fragments.

Despite major progress in our understanding of the pathogenesis of neurodegenerative diseases and the role of protein misfolding, effective treatments are still lacking. We hope that this special issue will help broaden the view of the problem and stimulate further research in the field.

*Alessio Cardinale* *Alessio Cardinale* *Roberto Chiesa* *Roberto Chiesa* *Michael Sierks* *Michael Sierks*

